# Practices, strategies, and challenges in antibiotic treatment and prevention of antimicrobial resistance from the perspectives of Romanian community pharmacists and general practitioners: a Goffman-inspired qualitative interview study

**DOI:** 10.3389/frabi.2024.1439688

**Published:** 2024-09-04

**Authors:** Lavinia Bianca Balea, Stinne Glasdam

**Affiliations:** ^1^ Faculty of Medicine, Lund University, Malmö, Sweden; ^2^ Integrative Health Research, Department of Health Sciences, Faculty of Medicine, Lund University, Lund, Sweden

**Keywords:** antimicrobial resistance (AMR), interview study, pharmacists, Goffman theory-inspired, general practitioners

## Abstract

**Introduction:**

Antimicrobial resistance (AMR) represents a persistent and ascensive global threat influenced by antibiotic misuse and overuse. In the Romanian context, patterns of antibiotic consumption and resistance within the healthcare system are marked in the red scenario on the European map. General practitioners and pharmacists, among others, play a major role in stewardship towards AMR.

**Aim:**

To explore the practices, strategies, and challenges in antibiotic treatment and prevention of antimicrobial resistance from the perspectives of Romanian community pharmacists and general practitioners.

**Method and Materials:**

Semi-structured interviews were conducted with six general practitioners and five community pharmacists in Romania from January to March 2024. An inductive, Goffman theory-inspired thematic analysis was conducted, inspired by Braun and Clarke’s thematic analysis method, consisting of familiarization with the data, iterative coding guided by theoretically inspired questions, and identification and refinement of (sub)themes. Goffman’s theory of social interaction, focusing on the concepts of front stage, backstage, and roles, guided the analytical questions.

**Results:**

The results were presented in three overarching themes: ‘Knowledge acquired backstage to support challenges and performance on front stage’, ‘Adapting roles and performances on the front stage: A mix of structured and twisted acts’, and ‘Interprofessional Collaboration: A latent part in the play’s roles and performances’. Professionals prepared their understandings of AMR and antibiotics backstage, with an awareness of the challenges rooted in the Romanian context. The front stage scenario evolved from structured antibiotic performances led by AMR strategies with compliant actors to challenging performances influenced by actors which changed the course of performances and intended AMR strategies. The revealed competition between general practitioners and pharmacists further complicated antibiotic use and AMR-related performances.

**Conclusion:**

The Romanian socio-political system influenced the course of antibiotic treatment and the professionals’ intended antibiotic related practices and AMR strategies. The study showed a theory-practice gap in health professionals’ practices, leading to limited strategy integration towards AMR and increased antibiotic use. The study underscores the need for context-specific policies and interventions to minimize identified gaps.

## Introduction

A revolutionary discovery marked the course of medicine in 1928 through the fortunate finding of the first molecule with the potential to kill bacteria – penicillin – known as the first antibiotic (Lobanovska and Pilla, 2017), opening the path to the ‘golden era’ of antibiotics. This came to an end due to the emerging antimicrobial resistance (AMR) (Aslam et al., 2018). AMR includes resistance to antibiotics, antifungal, antiviral, and antiparasitic agents by microorganisms (World Health Organization, 2023). The concept of AMR specifically refers to antibiotic resistance (World Health Organization, 2020). Bacterial AMR is when antibiotics become ineffective in treating infectious diseases and is the result of genetic mechanisms developed by bacteria (World Health Organization, 2023). The ability of bacteria to transfer resistance to another bacteria through horizontal gene transfer, understood as the process of genetic exchange between bacteria, is a key factor in the widespread dissemination of bacterial AMR, making it a significant public health concern (ReAct, 2020). AMR dissemination, driven by irrational antibiotic use (World Health Organization, 2023), creates complex issues impacting environmental, human, and animal health, with significant societal costs (Aslam et al., 2018; Habboush and Guzman, 2024). Understanding AMR requires both national and global perspectives.

In 2019, antibiotic-resistant bacterial strains were linked to 4.95 million deaths globally, with 1.27 million being direct causes (Murray et al., 2022). This threat surpasses that of HIV and malaria, making AMR one of the ten most critical global health issues according to the World Health Organization (2019). Economically, AMR is projected to cost $100 trillion by 2050, affecting healthcare costs, labour, global trade, and poverty (World Bank, 2017). By 2030, AMR may push 24 million people into extreme poverty and cause 10 million deaths annually by 2050 (World Bank, 2017). Humanity faces a ‘post-antibiotic’ era where basic infections may become incurable, threatening sustainable development goals (Jasovský et al., 2016). AMR is described as a ‘dystopian crisis,’ indicating severe societal impacts, or an ‘apocalyptic crisis,’ involving events that lead to catastrophic global changes, underscoring the critical role of antibiotics (Hansson and Brenthel, 2022).

Tackling the complexities of AMR requires intersectoral action as it is an issue stemming from multiple sectors, with no clear-cut solution (Dadgostar, 2019). The agricultural industry, biological factors, globalization, and climate change amplify the AMR burden by facilitating the spread of resistant bacterial strains (Dadgostar, 2019; United Nations Environment Programme, 2023). The pharmaceutical industry’s focus on profit-driven promotion of antibiotics, lack of interest in developing new antibiotics and environmental pollution also perpetuate AMR (Machowska and Lundborg, 2018; Kotwani et al., 2021; World Health Organization, 2022). Healthcare represents a major contributor to AMR through the misuse and overuse of antibiotics (Dadgostar, 2019). Globally, human antibiotic use showed an abrupt upward curve, with a 65% increase between 2000-2015 (Klein et al., 2018). Around 80-90% of antibiotic consumption is attributed to outpatient care (UK Health Security Agency, 2023). Moreover, many antibiotic prescriptions may be unnecessary due to the viral nature of infections (Centers for Disease Control and Prevention, 2022; McKay et al., 2016).

The current study offers a national perspective, focusing on Romania, a country marked in the red zone on the European AMR map (European Public Health Alliance, 2017). Romania registers one of the highest European rates of AMR (World Health Organization, 2020). The situation within the Romanian context may be more critical than the actual data suggests, as there is a lack of reporting systems and research (World Health Organization, 2020). Romania faces unique AMR challenges, including high antibiotic consumption and resistance rates, insufficient One Health action plan implementation, and cultural factors promoting irrational use (World Health Organization, 2020; Directorate-General For Health And Food Safety, 2022). The Romanian healthcare system has Europe’s highest community antibiotic consumption, worsened by limited awareness among healthcare providers and the public, leading to high self-medication and inappropriate use (European Centre for Disease Prevention and Control, 2022; World Health Organization, 2020).

According to Romanian legislation, antibiotics require a medical prescription, except in emergencies when pharmacists can dispense them without one (Ghiga and Lundborg, 2016). Smith (2015) argues that AMR is a social problem driven by the injudicious use of antibiotics and needs a renewed social approach. Thus, addressing AMR in Romania necessitates targeting the main actors involved in its dissemination. Among others, community pharmacists and general practitioners (GPs) significantly influence AMR stewardship through their knowledge, behavior, and external influences (Bishop et al., 2019; Hawes et al., 2020). Diagnostic uncertainty and patient factors contribute to inappropriate antibiotic prescribing and dispensing (Sijbom et al., 2023). Limited attention has been given to understanding the practical challenges and strategies used by these key antibiotic stewards in Romania. Few studies have explored Romanian community pharmacists’ and family doctors’ perceptions on AMR and their roles in addressing it. These studies identify systemic barriers, such as lack of diagnostics, inadequate regulations, and a limited GP workforce, exacerbating AMR. A weak regulatory environment and negative incentives push health professionals to the limits of the law. Potential improvements include a collaborative approach and more investment in educational interventions for pharmacists and the public (Ghiga and Lundborg, 2016; Ghiga et al., 2023). Additionally, self-medication is prevalent in Romania (Damian et al., 2014), similar to e.g., Greece and Cyprus (Lescure et al., 2018). Gaining a deeper understanding of pharmacists’ and GPs’ practices and strategies towards AMR and their challenges in practice regarding antibiotic treatment represents a starting point for future interventions that can be better adapted to the needs of the Romanian context. From a Goffmanian perspective, this study aimed to explore practices, strategies, and challenges in antibiotic treatment and prevention of AMR from the perspectives of Romanian community pharmacists and GPs.

## Theoretical framework

The empirical material was analyzed through the lens of Goffman’s dramaturgical perspective on social interaction (Goffman, 1990). Goffman used metaphorical concepts, where actors take different roles and adopt various strategies to act and interact based on the context in which they perform. The different roles are shaped to portray a desired image. The current study was inspired by the concepts of front stage, backstage, and role (Goffman, 1990).

Goffman (1990) conceptualized the actors’ context as unfolding on different stages, such as the front stage, representing the ‘spotlight’ context of a more restrictive nature since the actors know that an audience is watching them, maintaining a desirable public image by adhering to standards and conventions. Backstage represents the area ‘behind the front stage scenes’ where the actors can drop their public persona and set off the performance, representing a setting of preparation for the front stage performance (Goffman, 1990). Goffman identified various roles adopted by actors. The role of duty is assumed out of necessity, the attachment role is taken due to preference, the embracement role is voluntarily chosen, and the distancing role is adopted by those avoiding specific roles (Goffman, 1990).

The current study focused on how the actors performed front stage and backstage, together with whom and how. Goffman’s theory could contribute to understanding how the roles and social interactions unfolded between healthcare professionals and other actors from healthcare professionals’ perspectives. The theory also had the potential to focus on how challenges met by professionals influenced their practices and strategies and how challenges might influence their roles and ways of acting. In this study, the *front stage* represents the formal, visible actions of GPs and pharmacists, such as their interactions with colleagues and patients related to strategies on antibiotic use and prevention of AMR. These performances are shaped by professional expectations and social skills, which the analysis also focuses on. The *backstage* represents the more private and informal aspects of professionals’ work, away from the public, where professionals prepare for their front stage roles, develop strategies, etc. Overall, the study attempts to uncover how interactions between front stage and backstage behaviors might influence antibiotic stewardship. By connecting the theoretical concepts through construction analytical research questions and aligning them with the study’s aim, it provides a comprehensive framework for understanding the various interactions between key factors that influence antibiotic use and AMR.

## Materials and methods

A qualitative research methodology was employed to explore antibiotic treatment and AMR prevention in Romania (Dahlgren et al., 2019). Semi-structured interviews provided in-depth insights from participants’ perspectives (Kvale and Brinkmann, 2015). Data were analyzed using thematic analysis (Braun and Clarke, 2006) and Goffman’s dramaturgical theory (Goffman,1990). The study, conducted in urban Romania, covered both small municipalities (<70,000 inhabitants) and larger ones (>150,000 inhabitants).

### Sampling

The inclusion criterion was community pharmacists and GPs practising in Romania’s urban areas. There were no exclusion criteria.

In the first phase, convenience sampling was used. Three gatekeepers contacted known pharmacists and GPs to participate in the study, which resulted in seven included participants. Gatekeepers were selected from the first author’s professional networks. In addition, the first author contacted pharmacists and GPs directly at their workplaces, resulting in three included participants. One participant was recruited through snowball sampling, recommended by one participant. In total, eleven participants participated in the interview study, see **Table 1**. This was in line with Kvale and Brinkmann (2015), who describe that qualitative interview studies often consist of 15 +/- 10 participants. We assessed that our study achieved data saturation as no new information emerged after the eleventh interview. Once the participants expressed interest, they were provided with both oral and written information about the study.

**Table 1:** Characteristics of participants (*n* = 11).

	General Practitioners (n = 6)	Pharmacists (n = 5)
Gender		
Male	1	–
Female	5	5
Age years (average)		
	43-71 (63)	29-32 (30)
Years in the profession years (average)		
	3-45 (30)	6-7 (7)

### Collection of empirical material

A semi-structured interview guide was developed to support the interviews, structured upon three main topics: patients’ requests for antibiotics, the influence of guides and employed strategies, and collaboration between professionals. A pilot interview was conducted before the interview process to assess the suitability of the interview guide to train the interviewer’s technique (Kvale and Brinkmann, 2015). The pilot interview helped refine the interview guide, as it was too structured and the pre-formulated questions stopped the narratives more than it supported them. Therefore, the guide was revised to be shorter and more flexible, organized in themes with supportive keywords, allowing for a more spontaneous flow led by participants’ responses and probing questions. Moreover, some of the initial questions were leading participants in a certain direction, which was an important consideration to avoid when refining the interview guide.

Both in-person and online interviews were conducted in Romanian. Interviews were planned with the participants, considering their availability and preferences. The online interviews were conducted on an encrypted video platform, Teams. To foster rapport in all interviews, the interviewer engaged participants beforehand to inform about the study’s purpose and presented herself to encourage open responses. Efforts were made to create a comfortable environment to mitigate ‘social desirability’ and promote honest discussions about AMR challenges and practices. In-person interviews, conducted at community pharmacies and GPs’ offices, also provided the researcher contextual insights. The same interview guide was used for both online and face-to-face interviews to ensure consistency in data collection.

The interviews took place between 10 January and 7 March 2024. A video camera was used during the four online interviews to create a better contact with the participants and foster trust in the interview process. However, only the audio was recorded using an external recorder without an internet connection. The seven face-to-face interviews were conducted at the community pharmacies and GPs’ offices and recorded on an external device. The interview durations ranged from 19 to 70 minutes (average duration: 39 minutes) due to the fact that it takes different time for people to tell their story. However, the shortest interview was interrupted due to a duty call.

### Ethical considerations

The current study followed the ethical principles of the Helsinki Declaration (World Medical Association, 2013). The ethical considerations were reflected upon from the initial phases of the study. The principle of autonomy was followed by an informed consent and information letter, through which the participants were accurately informed about the purpose of the research and the right to withdraw at any moment without any explanation (Dahlgren et al., 2019). To ensure that the participants understood the details and the terms in the informed consent form, before the interview, all the information was presented again, and verbal consent was obtained, followed by written informed consent. All participants were informed that data were collected, stored, and analyzed in line with the Helsinki Declaration and General Data Protection Regulation practices. All data were anonymized and kept inaccessible to anyone other than the researchers. In publications, the study maximizes anonymity by removing personal names and place names.

### Analytical strategy

A latent thematic analysis, methodically inspired by Braun and Clarke (2006) and theoretically inspired by Goffman (1990), was conducted, to shape a narrative from the empirical material. This method allowed the researcher to be flexible in the analysis and to identify patterns of meanings regarding the explored phenomena. An inductive process was employed, going back and forth, following Braun and Clarke’s (2006) six analytical steps.

First, all interviews were transcribed and anonymized. Second, the transcriptions were read several times to familiarize with the empirical material. Next, the coding process was initiated by following an iterative process using following theory-inspired analytical questions:

- From GPs’ and pharmacists’ perspectives, how did the professionals and patients (inter)act regarding antibiotic treatment, and where? (Performance, strategies, front stage, backstage).
- Which roles did professionals have in encounters with patients related to antibiotics and AMR, and how did they perform? (Performance, strategies, roles, front stage).
- Which roles did professionals have in encounters with other professionals related to antibiotics and AMR, and how did they perform? (Performance, strategies, roles, front stage, backstage).

The fourth step consisted of construction for sub-themes and themes by finding a pattern through the generated codes. Furthermore, the potential sub-themes and themes concerning the dataset were reviewed by filtering them through the critical questions proposed by Braun and Clarke (2006). This process emerged in the fifth step of defining and naming singular sub-themes and themes, which directly addressed the aim and shaped a coherent narrative from the empirical material. The analytical process is exemplified in **Table 2**. The last phase of the analysis was writing the results during dialectic processes between the two authors. *Quotes* from the empirical material were translated into English and used as an illustration of the analysis.

**Table 2:** Examples of the analytical process.

Example Quotes	Codes	Subthemes	Theme
“So vis-à-vis resistance, nowadays, medicine is evidence-based [...] If I suspect microlithiasis, which predisposes to infection, the first step is not the antibiotic. The first step is a urine exam. Or if the patient has respiratory symptoms and has a fever, the first thing is the test” (GP1)	Displaying front stage rationality in performances	Performances and roles guided by tools to make the invisible visible	Adapting roles and performances on the front stage: A mix of structured and twisted acts
“The diagnosis is based only on the patient's evaluation [...] depending on this you decide, you have no other way, so our experience is the most important” (GP4)	Using experience as a behind-the scenes guidance for front stageperformances		
“I dispense only in situations where I consider it necessary, or anyway I dispense a small amount” (Ph1)	Taking the role of responsible prescriber front stage	Dodging antibiotics worked as an AMR strategy in practice	
“I try not to prescribe for minor symptoms. I don't write them at first, so I try to postpone the prescription for two or three days” (GP5)	Taking the role of antibiotic safeguards through delayed performances		
“I had a child who had nasopharyngitis [...] I said let's try without [antibiotics], but mother didn't want to because she was afraid he was too feverish, and I gave in [...] I have nothing to do. If I don’t prescribe antibiotics, they move to another doctor” (GP2)	Conforming to patients’ demands in frontstage performances to maintain as customers	Shifting performances: professionals' roles changed when patients took the lead role	
“A patient asked for an antibiotic because he'd been coughing [...] I refused. He threatened to call the owner of the pharmacy. Under these pressures, I had to release” (Ph2)	Power dynamics influencing front stage performances		

## Results

The results consisted of three main themes, supported by subthemes, see **Table 3**.

**Table 3:** Themes and sub-themes.

Themes	Sub-themes
Knowledge acquired backstage to support challenges and performance on front stage	Antibiotic use and AMR set a difficult scene
	Gaining knowledge on antibiotics and AMR: muddling through a maze
Adapting roles and performances on the front stage: A mix of structured and twisted acts	Performances and roles guided by tools to make the invisible visible
	Dodging antibiotics worked as an AMR strategy in practice
	Shifting performances: professionals' roles changed when patients took the lead role
Interprofessional collaboration: A latent part in the play's roles and performances	Aligned forces and divided fronts: same-side versus opposite-side collaboration.
	Pharmacists-GPs’ role dynamics in the antibiotic stewardship: from collaboration to competition

### Knowledge acquired backstage to support challenges and performance on front stage

#### Antibiotic use and AMR set a difficult scene

As the performances unfolded in the antibiotic arena, pharmacists and GPs projected themselves to have the primary roles of experienced actors in the AMR issue. They acknowledged the threat of AMR as influential in their patient-related performances. Their insights showcased the contribution of both the professional and unprofessional actors in the development of AMR:

*“Yes, it seems to me that the resistance is quite high, at least in our country. I have seen some patients with analysis results, especially from urine culture, and they appear resistant to many antibiotics”* (Ph1)

*“We, as GPs, do not have antibiotics so strong to cause resistance, but uncontrolled amounts of underdosing are used for a day or two. After they feel better, patients don’t follow the prescription [ … ] so they develop antibiotic resistance”* (GP4).

Several pharmacists acknowledged that the broad-spectrum antibiotics use by the actors within the Romanian context might contribute to a higher resistance. Such a scenario might occur where no antibiotics would be effective for bacterial infections, determining unsuccessful and more complex performances. The pharmacists not only showed awareness about AMR but also expressed concern about the future of antibiotics and AMR:

*“Because that’s how resistance is created because broad-spectrum antibiotics are given and when we’re going to need it nothing will happen again, we take it for nothing”* (Ph4)

The GPs supported this understanding of AMR and admitted that the current scenario needs to unfold after a clear strategy, where antibiotics must be reserved for situations where they should be used. Finding themselves in a scenario where the antibiotics acted as a double-edged sword, the strategies for tackling AMR were dependent on the primary actors in the antibiotic arena:

*“This fight against antibiotics, that they are good when they need to be used. Yes, so to adopt evidence-based medicine”* (GP1)

Professionals considered resistance an ignored issue in the Romanian context as the current scenario lacks a rational pathway. Antibiotics served as scene setters for the involved actors, challenging their performances. Irrational antibiotic treatments could lead to a domino effect affecting the main actors involved and their performances due to less effective antibiotic treatments that lead to AMR, complicating the course of their performances. Ultimately, it would affect the audience as irrational antibiotic treatment by one actor can exacerbate AMR to the distant actors, leading to undesirable effects of less effective antibiotic treatments and persistent diseases:

*“There is a lot of antibiotic treatment given without thinking, there are also the big side effects”* (GP6)

Rather than having a strategy where patients’ bodies served as a natural reservoir of immunity to fight bacterial infections, the primary intent was to use antibiotics to cure infections directly. The actors found themselves in a scene where not only were the effects of AMR exacerbated, but it also led to more complex medical performances:

*“Patients, first, need to develop their natural resistance, which in our country does not exist. So, in our country, there is no such thing as taking vitamins or eating healthily. We have many shortcomings. Increasing immunity would be most important”* (GP4)

The antibiotics and AMR challenges rooted within the Romanian context shaped professionals’ performances. The revealed interactions between systemic factors and individual practices reflected the complexity of these issues in their everyday practice.

#### Gaining knowledge on antibiotics and AMR: muddling through a maze

Backstage, pharmacists and GPs used different strategies to gain knowledge about antibiotic treatment and AMR, reflecting their diverse understanding of antimicrobial stewardship. This backstage scenario illustrated how professionals managed their roles and information sources and integrated their knowledge to address the AMR issue.

The scenario for both professional groups was that they lacked guidelines, which in Goffman terminology could be regarded as a lack of a script for the performance, to describe and support their front stage performance in their meetings with infected patients:

*“Effectively, guidelines do not exist, there are guidelines on the use of antibiotics, but as far as I know only in hospitals”* (Ph1)

*“As GPs, we do not have some independent guidelines”* (GP6)

It became evident that pharmacists and GPs had distinct approaches and perspectives towards AMR and antibiotics, reflecting varying modes of thinking and interpretation. However, some GPs were aware of the existence of treatment guidelines related to the type of antibiotic and dosage recommendations, which they seldom used in their backstage preparation or in their front stage performances in the encounters with patients:

*“We have guides, of course. When we have a bacterial infection, then we go and follow the guidelines”* (GP5)

Pharmacists and GPs used different backstage strategies to learn about antibiotics and AMR. One strategy was to use the Internet to prepare their front stage acting, where they met the patients’ potential needs for antibiotics. This strategy could have resulted in a misguidance in the antibiotic treatment depending on the selection of reliable online sources:

*“When we have doubts, we usually consult the National Agency for Medicines and Medical Devices website or Mediately”* (Ph1)

*“We do our research, we know, we have Google”* (GP3)

Additionally, front stage strategies unfolded through interactions with colleagues, such as discussions and participation at conferences focused on the theme of antibiotics, which at the same time functioned as backstage preparation for encounters with patients. Some GPs attended as an audience for colleagues’ front stage performances in the form of oral research presentations and posters. Furthermore, collegial discussions allowed the professionals to stay updated about antibiotic treatment and management:

*“We go to congresses, then we orient ourselves”* (GP6)

*“If we have a certain case, we sit together with colleagues and search on a certain topic”* (Ph3)

Another backstage strategy was to read books and seek insights from drug manufacturers as reliable sources of knowledge. While this strategy might have offered professionals valuable insights to guide their performances, it might have also led to biassed information when consulting drug manufacturers as a source due to its primary intent to promote certain antibiotics:

*“There are the drug agendas in which we have updates and the promotion of the drugs from the part of manufacturing firms”* (GP1)

*“We have a book of pharmacotherapies in the pharmacy, which we check if we want to orient ourselves towards an answer”* (Ph3)

All in all, despite lacking a script for the performance due to deficient guidelines, the professionals oriented themselves to gather information about antibiotics and better construct their strategies for AMR. Their performance in staying up to date with AMR and antibiotics could be regarded as ‘navigating a maze’ due to the extensive amount of available information, requiring them to select their sources carefully.

### Adapting roles and performances on the front stage: a mix of structured and twisted acts

#### Performances and roles guided by tools to make the invisible visible

Some GPs contended that their clinical front stage performance was guided by testing bacterial aetiology of the infectious state of patients, giving them hints about ways of approaching the infections. GPs presented themselves as rational antibiotic stewards, guided by an evidence-based medical act, that led their actions in the right direction. Patients were passive actors in this performance, as they served the professionals with their bodies and their biological samples as an object of exploration:

*“So vis-à-vis resistance, nowaday, medicine is evidence-based [ … ] If I suspect microlithiasis, which predisposes to infection, the first step is not the antibiotic. The first step is a urine exam. Or if the patient has respiratory symptoms and has a fever, the first thing is the test”* (GP1)

Diagnostic tests for bacterial infections acted as prompts for professionals, enabling them to effectively navigate and align their actions towards the appropriate course of action.

However, pharmacists revealed that a particular pitfall of the GPs’ performances was not following a medical act based on prompts, conflicting with the GPs’ narratives:

*“So far, I have not met any lady who does urine tests with antimicrobial susceptibility tests. They said their GP didn’t tell them. That’s what they should do first thing, an antimicrobial susceptibility test”* (Ph3)

Sometimes, pharmacists’ front stage performances were guided by antibiograms presented by the patients to inform the right course of action. Within this act, the patient took the role of a health seeker searching for readily available solutions:

*“I asked the patient to show me if she had tested with the antibiogram and she had recent tests, then I decided I needed to help her”* (Ph2)

Asking for guidance from pharmacists instead of consulting GPs for an antibiotic prescription showcased that the actors needing antibiotic treatment or medical advice opted for the easiest way to fulfil their intended purpose. Using antibiograms to influence decision-making placed pharmacists in a scenario where they exceeded their main roles as dispensers and acted as prescribers.

Pharmacists and GPs primarily relied on their professional experiences as a tool when interacting with patients with potential bacterial conditions. GPs had dual roles as diagnosticians and therapists during their encounters with patients. Former clinical experience in antibiotic decisions was incorporated into their antibiotic decision-making in practice:

*“The diagnosis is based only on the patient’s evaluation [ … ] depending on this you decide, you have no other way, so our experience is the most important”* (GP4)

Beyond their experience, GPs used critical thinking to guide their diagnostician roles. They tried to better adapt to the scenario in the lack of clinical guidelines to fulfil the script or act as prompts. Many GPs utilized their cognitive tools, which allowed them to diagnose and treat the bacterial infections supported by previous clinical cases, which showcased a note of subjectivity in their performances:

*“A young man came with signs of prostate adenoma [ … ] I diagnosed acute prostatitis and gave antibiotics. It’s about intuitive knowledge, empirical, and rational knowledge, and sometimes none of them is pure”* (GP3)

Front stage, several pharmacists projected themselves in the roles of responsible dispensers, adhering to the script by dispensing antibiotics only when considered necessary or dispensing limited quantities:

*“I dispense only in situations where I consider it necessary, or anyway I dispense a small amount”* (Ph1)

At times, pharmacists conformed their dispensing practices to external expectations, aligning their actions with their officially defined and self-perceived duty roles. Front stage, pharmacists often acted as an extension of doctors’ prescriptions, which functioned as forced actions with no possibility of improvisation for pharmacists:

*“If the doctor prescribed in a certain way, I am obliged to dispense” *(Ph4)

Some pharmacists were in unusual roles when faced with unclear antibiotic prescriptions that challenged their front stage performances. This performance unfolded by transitioning from an informed professional to a knowledge seeker role by finding the most available alternatives to guide upon for a safe antibiotic treatment:

*“It happens that I don’t necessarily know at all the medication, the dosage, and then I look for the most rapid option to guide upon”* (Ph3)

Overall, professionals used different available tools to guide their performances, from diagnostic ones to readily available ones, such as practical tools. They needed to adapt their performances to the available resources, showcasing that improvisation related to antibiotics and AMR is also necessary to adapt to the deficient scenarios.

#### Dodging antibiotics worked as an AMR strategy in practice

By emphasizing the importance of responsible antibiotic treatment and AMR implications, pharmacists and GPs took the role of educators front stage through the rise of AMR-related informed citizens, with patients taking the role of learners:

*“I ask if tests were done before the doctor prescribed the antibiotic, especially when I see broad-spectrum antibiotics prescribed* [ … ] *I always explain to them what antibiotic resistance means and how important it is to take care how we administer antibiotics”* (Ph2)

*“I explain to patients how much the body’s resistance* [ … ] *to antibiotics has increased and that it would be best to use as little antibiotic as possible not to need very strong antibiotics in case of serious diseases”* (GP2)

Front stage, some GPs also took the role of antibiotic safeguards by avoiding prescribing antibiotics for minor symptoms and emphasizing the use of delayed prescriptions. They adapted their strategies to the AMR scenario, reflecting a commitment to following a rationale strategy of antibiotic stewardship:

*“I try not to prescribe for minor symptoms. I don’t write them at first, so I try to postpone the prescription for two or three days”* (GP5)

However, pharmacists’ narratives pointed out that GPs sometimes issued the exact antibiotic prescription for the same patient. This practice was adopted to avoid repetitive medical performances within the same storyline, showcasing a conflicting performance compared to the one declared. In such cases, the GPs’ actions position them in the role of irresponsible antibiotic stewards in relation to the political guidelines, prioritizing their convenience over the patients’ well-being regarding AMR:

*“A patient with urinary tract infection* [ … ] *I asked for his prescription, and he pulled out of the wallet four prescriptions with ciprofloxacin [antibiotic]* [ … ] *it didn’t seem necessary to me. I asked him why he had four prescriptions, and he said, ‘The doctor gave me* [ … ] *so I wouldn’t go too soon to ask him again for a prescription for this’”* (Ph3)

Pharmacists used stories in their front stage encounters to increase patient awareness regarding antibiotic treatment, taking the role of mentors. They persuaded patients by using pathos elements to achieve their intended strategy in attempting to change the way patients understood and acted about antibiotics and AMR in the long run:

“[Used oneself as an example] *When I had a cold and a sore throat, I could not swallow from aching pain, and I explained to them that I was not using antibiotics. And that a sore throat can also last three or four days”* (Ph4)

From professionals’ perspectives, the front stage performance often had pharmacists and GPs in leading roles and patients in the supporting roles. The professionals’ strategies unfolded by the planned performance in interactions with patients who were active learners in grasping the rational antibiotic treatment and resistance-related issues. This strategy not only had the desired results, as patients showed trust in the professionals’ acts, but also showcased that engaged leading actors led to successful AMR performances:

*“A patient came with a prescription for two antibiotics* [ … ] *I explained to him that they are broad-spectrum antibiotics and that, at first, should start taking some sucking tablets* [ … ] *the patient decided not to take antibiotics [ … ] he came back and said he is very thankful that I didn’t let him take antibiotics”* (Ph4)

*“I had good communication with a patient, a lady, who had many comorbidities and to whom I didn’t consider it necessary to write antibiotics and who had become very insistent initially. We recalled her in two days to see what to do”* (GP3)

Some pharmacists remarked younger patients often were open-minded actors, compared to the elderly ones to adapt to the proposed performance of following responsible antibiotic treatment:

*“It seems to me that patients under 40 years old are starting to realize it* [the AMR]. [ … ] [They] *also ask the pharmacist for advice”* (Ph2)

From the perspective of some pharmacists, AMR could be regarded as a generation problem, where the younger generation represented a hope for the rise of educated and correct-behaving AMR citizens in the upcoming generations.

#### Shifting performances: professionals’ roles changed when patients took the lead role

According to professionals, some patients had the power to challenge the leading roles of the professional actors in front stage performances. This power asymmetry shift transformed the encounters’ dynamics, with patients taking on the dominant role and professionals assuming a subordinate position. Some patients took the roles of self-diagnosticians and self-prescribers when encountering potential bacterial diseases rather than seeking medical advice and prescriptions from the leading actors. Professionals assessed that those patients often used left-over antibiotics from previous prescriptions or family or friends:

“[A patient said] *I took Nolicin* [left-over antibiotic] *without a prescription because I have urinary tract infection”* (Ph4)

*“I had a pregnant woman* [ … ] *who took tetracycline [left-over antibiotic]”* (GP3)

From professionals’ perspectives, those types of patients wanted to be prepared for the role of self-diagnosticians and self-prescribers by having a stock of antibiotics, challenging the AMR strategies of professionals:

*“The biggest difficulty in practice is the demand for antibiotic ‘to have it at home’”* (GP4)

*“Patient simply wanted an antibiotic to have it at home if a cold would follow”* (Ph2)

Professionals noted that patients’ roles often were guided by perpetuated misinformation about antibiotic treatments. These self-reliant performers distanced themselves from the medical setting and transitioned to informal seeking advice, taking the roles of independent knowledge seekers. This brought to stage a chaotic play with actors performing in conflicting roles, with diverse understandings and strategies to handle antibiotics and AMR:

*“The godmother of sources for patients usually is: ‘Well, the neighbour took it [the antibiotic] and said it worked’”* (Ph5)

*“Through bus stations, they only talk about what diseases they have and what treatments to take* [ … ] *in a diabetes treatment, they gave up insulin and took inulin* [carbohydrate from plants]. *The same happens with antibiotics, same confusions, recommendations”* (GP4)

Moreover, professionals noticed difficulties grasping the difference between viral and bacterial infections among those misinformed patients. These clueless actors on the front stage directed the play to one where professionals were more a part of the stage decor:

*“Many don’t make a difference* [ … ] *between bacterial and viral infections”* (Ph1)

*“They don’t distinguish between virus and bacteria* [ … ] *don’t even believe in them until they see them flying like flies”* (GP3)

From professionals’ perspectives, some patients turned to dishonesty to obtain antibiotics by taking advantage of online prescriptions issued on social media, such as WhatsApp and Facebook. Such instances unfolded with patients in the role of opportunists:

*“Now the WhatsApp method* [ … ] *they come with the bag from another pharmacy, I see that they have the antibiotic in the bag, and they ask again for an antibiotic”* (Ph3)

Despite professionals acknowledging the lack of need for antibiotic treatment, they sometimes surrendered to patients’ demands. It was about keeping the actors within the same performance in the present and in the future, as the system allowed patients to obtain antibiotics by going from one GP/pharmacy to another, finding one willing to address their demands:

*“I had a child who had nasopharyngitis* [ … ] *I said let’s try without [antibiotics], but mother didn’t want to because she was afraid he was too feverish, and I gave in* [ … ] *I have nothing to do. If I don’t prescribe antibiotics, they move to another doctor”* (GP2)

*“I had a patient who for any abdominal pain used Zinnat* [antibiotic] [ … ] *I tried to convince her that it wasn’t good, she insisted, and then I gave in and dispensed”* (Ph5)

Within the dynamics of these performances and from professionals’ perspectives, pharmacist-patient interactions unfolded with mutual low trust, with patients finding themselves in a defensive and demanding role and professionals in a compliant role. Patients often perceived pharmacists as simple providers of antibiotics, with GPs being perceived as having a more recognized role:

*“I checked the doses, and they were wrong. I told the patient* [ … ] *he asked me not to give my opinion about how the doctor prescribed as it’s none of my business”* (Ph2)

*“The patients are quite disciplined, they listen to my advice”* (GP2)

Power dynamics took the stage in other scenarios, further complicating professional-patient interactions. Some pharmacists encountered external pressure from pharmacy chiefs to dispense antibiotics to meet the expectations of the influential patients. The pharmacy chiefs acted as backstage directors, exerting pressure on the professionals to take the role of followers, ensuring that the performance unfolded to their (economic) benefit:

*“A patient asked for an antibiotic because he’d been coughing* [ … ] *I refused. He threatened to call the owner of the pharmacy. Under these pressures, I had to release”* (Ph2)

Overall, a significant plot twist unfolded as patients took the front stage. This intriguing scenario pointed to the influence of patients’ limited understanding and power dynamics on professionals’ performances, challenging their traditional roles.

#### Interprofessional collaboration: a latent part in the play’s roles and performances

##### Aligned forces and divided fronts: same-side versus opposite-side collaboration

In interactions, the same side professionals engaged in collaborative discussions that unfolded as a well-coordinated play. Pharmacists and GPs narrated that within the same groups of professionals, they shared cases, asked for advice, and offered support, contouring a balanced play led by respect and trust. Thus, scenarios showed that actors’ collaboration in antimicrobial stewardship depended on the hierarchical position and was sustained by the unity of the same professional branch:

*“Yes, I always consult with colleagues* [regarding antibiotic treatment] *especially with two colleagues who have more experience than me or also in the case of calculating doses, especially in children”* (Ph6)

*“It’s best to collaborate* [ … ] *we have a group of 10 GPs, and when something is not clear, I write on the group, and everyone gives their opinion”* (GP2)

However, some GPs found that communication was more difficult to maintain with their younger colleagues due to the differences in age and perceptions. A gap between younger and older generations of GPs emerged, where younger actors were more up-to-date with the new information regarding AMR and antibiotics. This gap resulted in an inverted knowledge hierarchy among GPs, where the relatively least experienced became the most knowledgeable in the field of antibiotics:

*“Those who are young, we don’t even know them* [ … ] *maybe they’re on the street but we don’t know who they are”* (GP3)

The interactions between the different professional groups emerged primarily out of necessity, revolving around practical matters such as antibiotic stocks, or confirming prescription details. The professional actors played their roles without taking active parts in addressing the critical issue of AMR. These actors often found themselves in a situation where a lack of staff led to limited communication. This led to making spontaneous decisions without a thorough analysis of the long-term effects on AMR with an embedded risk of automatism in decision-making:

*“GPs are also very busy, and it is a crisis of GPs because most of them are elderly, they retire, no new doctors come in* [ … ] *there are situations that require [to consult]. But otherwise, no, we don’t do it”* (Ph1)

*“I have consulted with pharmacists in relation to medicines stocks or drug substitutes”* (GP5)

Some pharmacists believed that the communication depended on the GPs’ personality, as in some cases, the GPs were willing to discuss antibiotic prescriptions. In other cases, according to some pharmacists, GPs became defensive when pharmacists corrected them in their initial prescriptions:

*“It depends on each doctor. Some are quite responsive. Others don’t take it too well when we call them to tell them that doses are not calculated correctly”* (Ph2)

Collaboration depended on the hierarchical professional structure, with opinions valued within the same group of professionals. Actors’ communication within the different hierarchical structures was driven by practical necessities around antibiotic treatment, not by strategic approaches to address AMR.

##### Pharmacists-GPs’ role dynamics in the antibiotic stewardship: from collaboration to competition

The backstage unfolded as an arena where the play was led by power asymmetries between the two professional groups, with each profession presenting itself as the performance’s central hero. Some GPs expressed frustration concerning pharmacists’ front stage performances when their decisions regarding antibiotic treatment were disregarded, with pharmacists taking the leading role and assuming that antibiotics were necessary for encounters with patients. This decision by pharmacists showcased that each professional group pursued their interests instead of prioritizing patients’ benefits in the long run:

*“I’m a bit angry with pharmacists because they also prescribed [individually] treatment* [ … ] *I was upset with many of them because they were saying ‘because the doctor didn’t want to prescribe’, but instead of saying ‘maybe she didn’t think it was necessary’* [ … ] *we should be on the same page”* (GP6)

However, the tipping point of the act was reached when GPs were forced to adapt their performances based on pharmacists’ misguided decisions, dispensing antibiotics based on patients’ commitment to obtain a prescription from GPs. Within this scenario, GPs revealed a perceived pressure to prescribe antibiotics and took on the roles of passive prescribers:

*“Pharmacists have been upsetting me for a long time. They generously gave antibiotics saying ‘I’ll give you today, but tomorrow bring me the prescription’* [ … ] *therefore must go ahead and prescribe”* (GP1)

On the other hand, some pharmacists revealed improper practices on the GPs’ side, placing them in a plot where they were constrained to act on the verge of the law. GPs acted ignorant about antibiotics, AMR, and the regulatory framework. The roles reversed, with pharmacists themselves taking the stage as responsible actors in the role of prescribers and positioning GPs in the roles of the negative actors:

*“It’s somehow frustrating that even the doctors don’t write a prescription, as they should know that according to the law, antibiotics are issued on a prescription. Yet, many patients come from the GPs and say that the doctor suggested coming directly and taking a certain antibiotic”* (Ph1)

From the perspectives of some pharmacists, GPs acted as lawbreakers by prescribing using the telephone and WhatsApp. Pharmacists acted as fellow offenders as they complied with these practices:

*“During the COVID pandemic, we accepted phone prescriptions on WhatsApp* [ … ], *but after the pandemic, the Ministry of Health stopped accepting phone prescriptions. Unfortunately, doctors have continued the same practice* [ … ] *I’m sure they are informed that they are no longer allowed to issue prescriptions like this”* (Ph4)

All in all, GP-pharmacist interactions revealed a level of conflict. This scenario depicted a competitive performance, with actors in the play taking on the role of the lead performer. Professionals within each group blamed one another for improper antimicrobial stewardship practices in an attempt to distance themselves from responsibility.

## Discussion

The discussions focus on three main findings, namely (1) The gap between the knowledge and practices of professionals, which reveals the differences between the declared knowledge regarding antibiotics and AMR, and how their practices unfolded on front stage in encounters with patients, (2) Power relationships affected how professionals handled antibiotics, exploring how power in professional-patient interactions changed the course of antibiotic performances, and (3) Hidden competition between pharmacists and GPs regarding antibiotics and AMR strategies, delving into the limited collaboration between pharmacists and GPs revealed from the findings.

The findings showed that pharmacists and GPs had different understandings of challenges related to antibiotics and AMR. These professionals displayed a broad medical and societal knowledge about antibiotics and AMR and presented themselves as rational actors following a pre-defined medico-political performance aligned with AMR stewardship. However, a gap emerged between the public face presented through declared and actual front stage practices and interactions with patients, which also influenced performances. This aligns with Goffman (1990), showing that actors often present a desirable face to safeguard their public image. Other studies also show that levels of knowledge related to antibiotic prescriptions of pharmacists and GPs are not correlated with accurate antimicrobial stewardship (Gajdács et al., 2020; Poss-Doering et al., 2020). This aspect emerges in the research literature as a ‘theory-practice gap’ as it is suggested that practice in antibiotic stewardship is influenced by the perceptions of healthcare professionals and external factors rather than by their knowledge (Gajdács et al., 2020; Poss-Doering et al., 2020; Ashiru-Oredope et al., 2021). The theory-practice gap is highlighted in other medical contexts dominated by neoliberal organizational structures, which focus on efficiency and financial interests over patient well-being (Glasdam et al., 2020). This aspect can be regarded as incorporated knowledge, where societal order becomes progressively embedded in peoples’ minds (Bourdieu, 1984). Such theory-practice gaps may exacerbate the spread of antibiotic-resistant bacteria, leading to prolonged hospitalizations and higher morbidity and mortality rates (Barker et al., 2017). Limited diagnostic capacity may lead to the malpractice of prescribing broader antibiotics, exacerbating AMR (Ghiga et al., 2023), which calls for development of guidelines, diagnostic opportunities, and education/training to support professionals’ practices (Lazure et al., 2023). The findings showed that the front stage performances of the professionals were influenced by the business facet of healthcare, contributing to the knowledge-practice gap. The pharmacists and GPs were situated in the light of ‘money-makers’ rather than acting as health-politically correct medical providers and carers about antibiotics and AMR, dispensing antibiotics without prescription or prescribed antibiotics when not necessary for fear of losing clients. Their primary intent was to ensure future coactions rather than prioritize the patient and AMR issue, where professional knowledge seemed to be subordinated financial interests. It may represent a structural barrier for professionals to meet their ideological-defined deontological roles (Ghiga and Lundborg, 2016). The gap between the ideology of being a healthcare professional and the actual reality, where professionals act as constrained actors to serve the interests of healthcare as a business, is consistent with previous literature (Saleh et al., 2021; Saliba-Gustafsson et al., 2021). In future research, it is important to explore the health consequences of economic interests in health services and the significance this holds for the development of AMR and related consequences.

Moreover, the findings showed that power relationships shaped the performances of all actors involved in the encounters. Also, patients had the power to change the course of performances as power struggles in the interaction between patients and professionals appeared to strain the performances. Traditionally, professionals lead healthcare performances and make the ultimate decisions regarding antibiotic treatments (Carlsson et al., 2023). Patients’ involvement in professionals’ decisions was forced, with patients resorting to any means to obtain antibiotics, such as dishonesty or leveraging their influence. This underscores the symbolic significance attributed to antibiotics within the cultural context, which shapes a system of beliefs associated with safety and healing (Bosley et al., 2022; Carlsson et al., 2023). Patients’ limited understanding of antibiotics and AMR often leads to increased expectations for antibiotic treatment, influencing pharmacists’ and GPs’ practices (Fletcher-Lartey et al., 2016; Kong et al., 2019). Consequently, patients also play a pivotal role in influencing antibiotic use and AMR stewardship (Kandeel et al., 2014; Wang et al., 2017).

The non-medical actors also influenced professionals’ antibiotic decisions in other scenarios. The patients confessed and trusted non-medical sources in their help-seeking behaviors. This aligns with other studies, showing that seeking advice about antibiotic treatment within personal networks is prevalent even in developed contexts like the United Kingdom, leading to a decrease in seeking medical advice (Ellis et al., 2019). In such scenarios, patients exercise agency in deciding on antibiotic treatment and choosing information sources, thereby influencing the course of professionals’ performance. The patients’ limited knowledge, revealed in the study according to professionals’ perspectives, shifted the symbolic power from healthcare professionals towards patients. In 2022, 39% of Europeans considered antibiotics to be effective against viruses, which is consistent with relatively low economic and education levels (European Commission, 2022). Moreover, antibiotics are linked with power and expertise in high power distance societies, where hierarchical levels influence patient-physician interaction, with the doctor holding primary authority (Deschepper et al., 2008). In Romania, there seems to be a substantial culture among patients to expect antibiotics, aligning with Broom et al. (2021) views in India. Studies show that patients’ pressure and expectations lead to an increase in GPs’ prescriptions of antibiotics and pharmacists’ dispensation of antibiotics without prescription (Dempsey et al., 2014; Zapata-Cachafeiro et al., 2019), often leading to increased unnecessary antibiotic prescriptions (Sirota et al., 2017; Kohut et al., 2020). However, there are contexts, like Sweden, where the patients have high levels of awareness regarding antibiotic treatments and high trust in the physician’s decision not to prescribe antibiotics (Vallin et al., 2016). Power relationships between professionals and patients can vary across different socio-economic and cultural contexts (Papadimou et al., 2022; Susilo et al., 2019), calling for further research on the impact of social inequality on antibiotic use/prescription and the AMR stewardship.

The power of patients raises questions about professionals’ roles beyond acting as an extension of the socio-political system in relation to AMR and antibiotic treatment. Borek et al. (2020) argue that systemic issues hinder AMR stewardship due to the influence of factors on individual, practical, community, and national levels. A systemic issue within the Romanian context is represented by the weak institutional capacity, with professionals considering that the state does not support the AMR stewardship through regular checks of law enforcement regarding issuing antibiotic treatments (Ghiga and Lundborg, 2016; Ghiga et al., 2023). It is important to reflect upon the symbolism of antibiotics in Romania and similar countries to understand its influence on patients’ behaviors and how it affects professional-patient relationships.

Furthermore, the study pointed to limited communication between the main actors involved in antibiotic stewardship, which is opposite to an Australian study, showing that GP-pharmacists collaborating as a team towards antimicrobial stewardship interventions can optimize the use of antibiotics within the primary care sector (Saha et al., 2021). The current findings pointed to a rather competitive nature of the supposed GP-pharmacist collaboration. Such collaboration may hinder opportunities to strengthen AMR stewardship, both in Romanian and other contexts, given the critical situation of AMR. This calls for future collaborative strategies nationally and internationally across professions and professional interests.

The interactions between Romanian pharmacists and GPs seemed to be formalized and driven out of necessity, taking the shape of a superficial act between the leading performers. A study conducted in Romania suggests that this limited collaboration stems from a low bridging capital, which could result in a weak information exchange related to antibiotics between the two professional groups (Ghiga and Lundborg, 2016). In a Norwegian study, pharmacists are perceived in a subordinate hierarchical position by both GPs and patients, acting as ‘GPs extended arms’ (Bergsholm et al., 2023). Bradley et al. (2018) describe these hierarchical differences as a ‘GP-pharmacist game’, where pharmacists adapt to the unwritten rules of the game to avoid conflicts, leading to further sustaining the traditional hierarchies and not consolidating the trust between the two professional groups. However, in Romania, pharmacists are not always subordinated to GPs in antibiotics prescriptions, as they also have the legal right to prescribe in emergency cases (Ghiga and Lundborg, 2016), leading to AMR-related issues. To address this issue, there needs to be clearly defined roles for who can prescribe antibiotics, and guidelines should be developed for how and when to prescribe them.

The GP-pharmacist age gap further contributed to the competitive nature of the two groups of professionals hindering AMR stewardship. This corroborates previous findings that show that age influences GP-pharmacist interactions, as more experienced physicians are less willing to collaborate with younger pharmacists (Van et al., 2011). To achieve good treatment outcomes and, at the same time, prevent AMR, it seems necessary to work for good collaborative relationships across professions. There are contexts, such as The Netherlands, Switzerland, and Belgium, where the pharmacist-GP collaboration is improved by establishing GP-pharmacist pharmacotherapy discussion groups (Macé et al., 2023). Professionals’ collaboration is embedded in the hierarchical structures within the Romanian and other contexts (Essex et al., 2023). This reveals a healthcare system where AMR stewardship may be hindered due to the cultural norms characterized by a high-power distance structure. However, a negative case analysis shows that the power distance may be lowered by sensitizing GPs about pharmacists’ potential expertise (Rieck, 2014). The current study’s findings pointed to the fact that the power distance may also be lowered by sensitizing pharmacists about GP’s potential expertise.

Finally, the current study has strengths and limitations. The qualitative empirical material was created through narratives of pharmacists and GPs about their practices, strategies, and challenges. In addition to complying with formal research ethics guidelines and laws, conducting the interviews required the ability to act ‘ethically on the spot’, where the interviewer must constantly be aware of both small and large signs indicating whether the interview was being conducted ethically and be able to respond appropriately (Øye et al., 2016). All participants shared their stories and expressed great satisfaction with the interview process, often verbally indicating their positive experiences afterward, which also witnessed good ethical interview practice. During one interview, an urgent incident occurred that required the interviewer’s immediate attention, resulting in the interview being promptly terminated. It is well-known that there is a difference between individuals’ narrated actions and the actual reality as not all the actions are apparent to the person acting, such as the influences of structural factors (Bourdieu, 1993). These narratives are limited to the perspectives of the interviewed professionals, also when it comes to their encounters with patients. Despite this, the interviewer enhanced credibility by clarifying and confirming details with participants during the interviews (Korstjens and Moser, 2018).

The results are based on a limited sample of pharmacists and GPs in Romania, which may restrict the transferability of the findings (Thomas and Magilvy, 2011). However, the study’s transferability was enhanced by providing rich, detailed data through ‘thick descriptions’ and by aligning findings with other studies (Korstjens and Moser, 2018). Convenience sampling, allowed for efficient recruitment and ensured participants’ comfort. Snowball sampling, used at a later stage, helped expand the sample size and reach individuals who might otherwise have been inaccessible. In hindsight, starting with snowball sampling alongside gatekeeper recruitment could have facilitated easier access to more participants. Determining when data saturation is achieved is always debatable and, from a philosophical standpoint, inherently impossible, as interviews are dynamic, with new questions and responses emerging throughout the process (Guest et al., 2006). Overall, the empirical material was deep and voluminous, and the iterative analysis process enhanced the results’ dependability (Forero et al., 2018). Additionally, the patients’ perspectives are not considered to gain a relational understanding of encounters with antibiotic treatment and AMR strategies, which is important to consider in future research.

Clear presentation of participant characteristics, study settings, and data collection methods, along with illustrative quotes, improved transparency and confirmability. Throughout the process, the first author critically reflected on her positionality as a pharmacist, considering how her assumptions might impact the study. The last author, while not a pharmacist or GP, brought extensive experience in AMR from a sociological perspective. Both authors maintained a focus on their pre-understandings, engaging in ongoing discussions and reflections on methodological and theoretical aspects. A reflexive journal with detailed notes supported these reflections. The theoretical perspective helped uncover participants’ underlying intentions and interpretations, while continually challenging immediate understandings of reality (Bourdieu and Wacquant, 1992) strengthened the study’s trustworthiness. Insider knowledge, as discussed by other researchers (Bourdieu et al., 1999; Burns et al., 2012; Bukamal, 2022), was both a strength, aiding in recruitment and rapport-building, and a potential source of pre-conceptions. The analytical lens based on Goffman’s social interaction minimized researchers’ preconceptions and shifted focus to a theoretical perspective, enhancing transparency and trustworthiness (Glasdam et al., 2024).

## Conclusion

This study contributed to theoretical knowledge related to the challenges that influence the practices of main antibiotic stewards in Romania and their endorsed strategies towards AMR. This study found that Romanian pharmacists’ and GPs practices, strategies, and challenges in the Romanian context were complex, and constructed around a socio-political system that affected the course of antibiotic treatment and the professionals’ intended strategies for AMR. From the perspective of professionals, patients also challenged health professionals’ practices, leading to an increase in antibiotic consumption and non-prevention of AMR. Antibiotics became the symbol of the play in professional performances, one where the vested interests of different actors were at the top of the performances. Pharmacists and GPs faced a theory-practice gap, as they frequently struggled to implement their strategies during patient encounters due to the business aspects, and the knowledge did not evolve in front stage performances, leading to irrational antibiotic use and possible AMR exacerbation. Although professionals declared the use of strategies in their practices, front stage performances revealed a limited integration of strategies towards AMR. The dispensation or prescription of antibiotics without an antimicrobial stewardship basis seemed a significant concern for public health in Romania. It was evident that context played an important role in antibiotic use and AMR prevention strategies.

This study underscored the need for context-specific policies and better-tailored interventions to minimize the identified gaps. These interventions and policies can better align to the context’s needs through the theoretical understanding of the AMR situation related to practices, strategies used and encountered challenges within the Romanian context. Antibiotic use and AMR practices, strategies, and challenges were influenced by different actors, which policymakers must take into consideration for policy efficiency. The study also highlighted the crucial role of improved communication between professionals, emphasizing the need for collaborative efforts towards AMR stewardship.

However, the article represents a scratch on the surface in filling the existent gaps within the Romanian context. The current study calls for future research to explore the social and economic factors influencing antibiotic use to gain a deeper understanding of the knowledge-practice gap revealed within this study and the potential influences of other contextual factors. The issue of self-medication among patients and the prevalence of misinformation regarding antibiotic resistance remain largely unexplored. The question of why some patients perceive antibiotics as a universal cure for their health issues, as regarded from the professionals’ perspectives, remains unanswered. Additionally, including the efforts of those in the higher political sphere to explore their awareness and perceived roles in AMR could provide valuable insights. Further studies on how to enhance communication between different disciplines of professionals seem important in developing and implementing AMR strategies in practice. Furthermore, the study invites policy-makers to prioritize the issue of theory-practice gap on their agendas, developing new targeted strategies to ensure that healthcare providers have the necessary support and resources for antibiotic stewardship. This also calls for future evaluative studies on the implementation of these strategies. Raising awareness of this issue at both the policy and clinical level is essential for driving systemic changes that can effectively mitigate the challenges associated with AMR.

## Data Availability

The datasets presented in this article are not readily available because data can not be shared outside the research team. Requests to access the datasets should be directed to lavinia.balea94@gmail.com.
